# Supplementary material for: From sequence to enzyme mechanism using multi-label machine learning
Source: BMC Bioinformatics. 2014 May 19;15:150. doi: 10.1186/1471-2105-15-150 (PMC4229970; doi:10.1186/1471-2105-15-150)
Supplement: Additional file 2 — Java code of ml2db. Additional file ml2db_code.tar.gz contains the Java source code to run the multi-label machine learning experiments and save the results to database. The code’s Javadoc is included. [file 1471-2105-15-150-S2.zip › additional file 2/ml2db/ml2db/doc/main/ml2db/al/utility/globaldistance/GlobalDistanceMetric.html]

GlobalDistanceMetric


JavaScript is disabled on your browser.


- Overview
- Package
- Class
- Use
- Tree
- Deprecated
- Index
- Help

- Prev Class
- Next Class

- Frames
- No Frames

- All Classes

- Summary:
- Nested |
- Field |
- Constr |
- Method

- Detail:
- Field |
- Constr |
- Method


main.ml2db.al.utility.globaldistance

## Class GlobalDistanceMetric

- java.lang.Object
- - main.ml2db.al.utility.UtilityMetric
  - - main.ml2db.al.utility.globaldistance.GlobalDistanceMetric

- Direct Known Subclasses:
  :   GlobalDistanceWithConfidenceMetric

  ---

    

  ```
  public class GlobalDistanceMetric
  extends UtilityMetric
  ```

  Positioning metric: a metric that considers positively if an instance is
  close to other unlabelled instances and distant from other labelled
  instances. It needs a measure of distance between an instance and other
  labelled (L) and unlabelled (U) instances. Formula: Positioning(i) = lambda
  Sum dist(i,l) - (1-lambda) Sum dist(i,u) where lambda is a balancing factor,
  i is the target instance, l is each labelled instance and u is each
  unlabelled instance. And dist(x,y) is a function that returns a numerical
  representation of "distance" between instances x and y (for example,
  Euclidean distance). The distance between any two instances (set of
  attributes) is stored in memory for reuse.

  Version:
  :   7 Jul 2011

  Author:
  :   Luna De Ferrari ldf(at)st-andrews.ac.uk

- - ### Field Summary

    Fields

    | Modifier and Type | Field and Description |
    | --- | --- |
    | `static java.lang.String` | `NAME` |
    | `static double` | `STANDARD_BALANCE` An equal (50%) weight between distance to labelled and unlabelled instances. |
  - ### Constructor Summary

    Constructors

    Constructor and Description

    `GlobalDistanceMetric()`
  - ### Method Summary

    Methods

    | Modifier and Type | Method and Description |
    | --- | --- |
    | `double` | `getBalance()` |
    | `DistancesCalculator` | `getDistanceCalculator()` |
    | `double` | `getDistances(weka.core.Instance instance, int labellingRole)` |
    | `IdMultiLabelInstances` | `getLabelled()` |
    | `double` | `getLabelledDistances(weka.core.Instance instance)` |
    | `java.lang.String` | `getMetricName()` |
    | `GlobalPositionStore` | `getPositionsStore()` |
    | `double` | `getUnlabelledDistances(weka.core.Instance instance)` |
    | `double` | `getUtility(mulan.classifier.MultiLabelLearner trainedLearner, IdInstance instance)` Gets a measure of how distant the instance is from other unlabelled instances (good if it's close to them) and labelled instances (bad if it's close to them). |
    | `void` | `initialise()` |
    | `void` | `initialise(IdMultiLabelInstances labelled, IdMultiLabelInstances unlabelled)` |
    | `boolean` | `isEmptyLabelledSet()` |
    | `static void` | `main(java.lang.String[] args)` |
    | `void` | `refresh(IdMultiLabelInstances labelled, IdMultiLabelInstances unlabelled, IdInstance instanceLabelled)` Refresh the set of labelled and unlabelled instances |
    | `void` | `serializeUnlabelledPositions(int arffId)` Before serialisation, fill global positions. |
    | `void` | `setDebug(boolean debug)` |
    | `void` | `setEmptyLabelledSet(boolean emptyLabelledSet)` |
    | `void` | `setInstances(IdMultiLabelInstances labelled, IdMultiLabelInstances unlabelled)` |
    | `void` | `updateDistances(weka.core.Instance instanceLabelled)` |

    - ### Methods inherited from class main.ml2db.al.utility.UtilityMetric

      `getUnlabelled, setUnlabelled`
    - ### Methods inherited from class java.lang.Object

      `equals, getClass, hashCode, notify, notifyAll, toString, wait, wait, wait`

- - ### Field Detail


    - #### STANDARD\_BALANCE

      ```
      public static final double STANDARD_BALANCE
      ```

      An equal (50%) weight between distance to labelled and unlabelled
      instances.

      See Also:
      :   Constant Field Values


    - #### NAME

      ```
      public static final java.lang.String NAME
      ```

      See Also:
      :   Constant Field Values
  - ### Constructor Detail


    - #### GlobalDistanceMetric

      ```
      public GlobalDistanceMetric()
      ```
  - ### Method Detail


    - #### getBalance

      ```
      public double getBalance()
      ```


    - #### getDistanceCalculator

      ```
      public DistancesCalculator getDistanceCalculator()
      ```


    - #### getDistances

      ```
      public double getDistances(weka.core.Instance instance,
                        int labellingRole)
      ```


    - #### getLabelled

      ```
      public IdMultiLabelInstances getLabelled()
      ```


    - #### getLabelledDistances

      ```
      public double getLabelledDistances(weka.core.Instance instance)
      ```


    - #### getMetricName

      ```
      public java.lang.String getMetricName()
      ```

      **Specified by:**
      :   `getMetricName` in class `UtilityMetric`


    - #### getPositionsStore

      ```
      public GlobalPositionStore getPositionsStore()
      ```


    - #### getUnlabelledDistances

      ```
      public double getUnlabelledDistances(weka.core.Instance instance)
      ```


    - #### getUtility

      ```
      public double getUtility(mulan.classifier.MultiLabelLearner trainedLearner,
                      IdInstance instance)
      ```

      Gets a measure of how distant the instance is from other unlabelled
      instances (good if it's close to them) and labelled instances (bad if
      it's close to them).

      **Specified by:**
      :   `getUtility` in class `UtilityMetric`

      Parameters:
      :   `balance` - if > 0.5, the distance from labelled instances counts more
          than the closeness to many unlabelled instances. The other way
          round if < 0.5.
      :   `distancesFromLabelled` - the distances between this instance and each labelled instance
      :   `distancesFromUnlabelled` - the distances between this instance and each unlabelled
          instance

      Returns:
      :   a value representing the position of this instance in relation to
          other labelled and unlabelled instances.


    - #### initialise

      ```
      public void initialise()
      ```

      **Specified by:**
      :   `initialise` in class `UtilityMetric`


    - #### initialise

      ```
      public void initialise(IdMultiLabelInstances labelled,
                    IdMultiLabelInstances unlabelled)
      ```


    - #### isEmptyLabelledSet

      ```
      public boolean isEmptyLabelledSet()
      ```


    - #### refresh

      ```
      public void refresh(IdMultiLabelInstances labelled,
                 IdMultiLabelInstances unlabelled,
                 IdInstance instanceLabelled)
      ```

      **Description copied from class: `UtilityMetric`**

      Refresh the set of labelled and unlabelled instances

      **Specified by:**
      :   `refresh` in class `UtilityMetric`


    - #### serializeUnlabelledPositions

      ```
      public void serializeUnlabelledPositions(int arffId)
      ```

      Before serialisation, fill global positions.


    - #### setDebug

      ```
      public void setDebug(boolean debug)
      ```


    - #### setEmptyLabelledSet

      ```
      public void setEmptyLabelledSet(boolean emptyLabelledSet)
      ```


    - #### setInstances

      ```
      public void setInstances(IdMultiLabelInstances labelled,
                      IdMultiLabelInstances unlabelled)
      ```


    - #### updateDistances

      ```
      public void updateDistances(weka.core.Instance instanceLabelled)
      ```


    - #### main

      ```
      public static void main(java.lang.String[] args)
      ```


- Overview
- Package
- Class
- Use
- Tree
- Deprecated
- Index
- Help

- Prev Class
- Next Class

- Frames
- No Frames

- All Classes

- Summary:
- Nested |
- Field |
- Constr |
- Method

- Detail:
- Field |
- Constr |
- Method
